# Human papillomavirus E7 protein induces homologous recombination defects and PARPi sensitivity

**DOI:** 10.1007/s00432-023-05511-6

**Published:** 2024-01-23

**Authors:** Siqi He, Ao Wang, Jing Wang, Zizhi Tang, Xiaojun Wang, Danqing Wang, Jie Chen, Cong Liu, Mingcai Zhao, Hui Chen, Liang Song

**Affiliations:** 1 Key Laboratory of Birth Defects and Related Diseases of Women and Children (Ministry of Education), Department of Gynecology and Obstetrics, Meishan Women and Children’s Hospital, West China Second University Hospital, Sichuan University, Chengdu, 610041 People’s Republic of China; 2 Department of Clinical Laboratory, Suining Central Hospital, Suining, 629000 People’s Republic of China

**Keywords:** HPV, DNA repair, Checkpoint, PARP inhibitor, cervical cancer

## Abstract

**Purpose:**

Cervical cancer is a common gynecological malignancy, pathologically associated with persistent infection of high-risk types of human papillomavirus (HPV). Previous studies revealed that HPV-positive cervical cancer displays genomic instability; however, the underlying mechanism is not fully understood.

**Methods:**

To investigate if DNA damage responses are aggravated in precancerous lesions of HPV-positive cervical epithelium, cervical tissues were biopsied and cryosectioned, and subjected to immunofluorescent staining. Cloned HA-tagged E6 and E7 genes of HPV16 subtype were transfected into HEK293T or C33A cells, and indirect immunofluorescent staining was applied to reveal the competency of double strand break (DSB) repair. To test the synthetic lethality of E7-indued HRD and PARP inhibitor (PARPi), we expressed E7 in C33A cells in the presence or absence of olaparib, and evaluated cell viability by colony formation.

**Results:**

In precancerous lesions, endogenous DNA lesions were elevated along with the severity of CIN grade. Expressing high-risk viral factor (E7) in HPV-negative cervical cells did not impair checkpoint activation upon genotoxic insults, but affected the potential of DSB repair, leading to homologous recombination deficiency (HRD). Based on this HPV-induced genomic instability, the viability of E7-expressing cells was reduced upon exposure to PARPi in comparison with control cells.

**Conclusion:**

In aggregate, our findings demonstrate that HPV-E7 is a potential driver for genome instability and provides a new angle to understand its role in cancer development. The viral HRD could be employed to target HPV-positive cervical cancer via synthetic lethality.

## Introduction

Cervical cancer is an aggressive gynecological malignancy. The incidence of cervical cancer has been increasing in recent years, despite prospective decrease with the protection of HPV vaccination. More than 90% of cervical cancers are associated with high-risk human papillomavirus, which encodes two critical oncoproteins, E6 and E7 (Li et al. 2017; Yugawa and Kiyono 2009). E6 is reported to promote p53 degradation, while E7 binds to DNMT1 and increases DNA methylation, leading to inactivation of CpG island (Dueñas-González et al. 2005; Leonard et al. 2012; Burgers et al. 2007). In addition, cervix cancer display genomic instability that could be attributed to E7, the expression of which increases γH2AX, the marker of DSB repair (Fan et al. 2013; Park et al. 2014).

In responsive to DSB, histone variant H2AX is phosphorylated at serine 139 (γH2AX) and appears in early phase of DDR, the herald of ATM and ATR-dependent checkpoint activation (Kinner et al. 2008). To elicit DSB repair, 53BP1 and BRCA1 coordinatively act at DNA ends to control the progression of DNA end resection that generates appropriate amount of single-strand DNA (ssDNA) to activate homologous recombination repair (HR). Replication protein A (RPA) binds ssDNA to form nucleofilament in a BRCA1-dependent manner, which subsequently attracts RAD51 recombination to promote sister chromatin exchange (Zeng et al. 2016). In the case of insufficient ssDNA production, 53BP1 drives the repair mechanism to non-homologous end joining (NHEJ) that is error-prone when compared with HR. The BRCA1-53BP1 pair determines the pathway choice between HR and NHEJ (Bunting et al. 2010), disturbance of which leads to mutagenic processes and rapid evolution of the cancer genome.

In recent years, a frontier anti-cancer therapy employing the synthetic lethality between HRD and PARP inhibitor has been applied successfully in breast and ovarian cancer (Harter et al. 2022; Fasching et al. 2021; Lau et al. 2022). Poly(ADP-ribose) polymerases (PARP) play important roles in base excision repair (BER), the repair mechanism in parallel of HR during genomic DNA replication (Hirota et al. 2022; Molla et al. 2020). PARP inhibition accumulates DSBs that obligates HR in non-cancerous cells, but causes overwhelming NHEJ activity in HRD cancer cells. Lethality is thereby induced due to chromosomal aberrance as the outcome of illegitimate end joining (Spiegel et al. 2021; Ma et al. 2023).

In the current work, we demonstrate the influence of E7 in DSB repair. This viral protein perturbs ssDNA processing and reduced RAD51-mediated HR. The impediment of DSB repair may contribute to the elevated level of endogenous DNA breaks in HPV + cervical epithelium. As per the HRD phenotype upon E7 expression, cervical cancer cells are hypersensitive to PARP inhibition. The synthetic lethality between HPV-induced HRD and PARPi may provide another therapy strategy that potentially benefits cervical cancer patients.

## Materials and methods

### Sample collections from patient

Cervical tissues were collected from West China Second University Hospital, Sichuan University. HPV positivity and subtypes were determined by the hybridization capture using HC2 High-Risk HPV DNA Test (QIAGEN, 5199–1220). RLU/CO value > 1.2 were dragonized as high-risk HPV positivity. All sample collections were informed to patients and consented by them, and were carried out in accordance with the Ethics Guidelines and Regulation of the West China Second University Hospital, Sichuan University. Patients signed the informed consent and agreed to use it for scientific research.

### Cell culture

HPV-negative cervical cancer cell lines (C33A) and human kidney epithelial cell line (HEK293T) were purchased from National Infrastructure of Cell Line Resource. RPE1-hTERT was gifted by the Genome Damage and Stability Centre, Sussex. Cells were cultured in DMEM (GIBCO) supplemented with 10% Fetal Bovine Serum (FBS, Hyclone) and 1%P/S (Penicillin and streptomycin, GIBCO). For transfection with Lipofectamine 3000® (Invitrogen, L3000015), cells were plated in 24-well plates and propagated to 80% confluency. Transfection was performed according to supplier’s instructions and cells harvested 72-h post-transfection.

### Plasmids

The open reading frames of HPV16 E6 and E7 were inserted into the EcoRI and NotI site of pLVX-IRES-ZsGreen1 vector with a N-terminal HA-tag, using NEBuilder® HiFi DNA Assembly Master Mix (NEB, E2621).

### Irradiation and chemical treatment

Custom-made X-ray machine (Wandong Ltd, Beijing) was used to conduct ionizing radiation at 1 Gray/min and dosages were depicted in respective experiments. Calibration of radiation dosage was carried out by manufacture annually. 2 μM chemotherapy chemical (CPT) was added to cells with confluence of 60%–70%.

### Immunofluorescence staining

Cells grown on cover glasses were fixed with 4% paraformaldehyde (PFA), then used 0.3% Triton X-100 to permeabilize. Or in some experiments cells were fixed and permeabilized with methanol:ethanoic acid (3:1) for 15 min. Cells were blocked for 30 min at 37 °C with 3% BSA in PBS supplemented with 0.2% Triton X-100. Blocked cells were then incubated for 40 min in 37 °C with diluted primary antibodies: Mouse anti-γH2AX (Millipore, 05–636), Mouse anti-ATM-pS1981 (Cell Signaling, 4526S), γH2AX Rabbit anti-53BP1 (Cell Signaling, 3428P), Rabbit anti-BRCA1-pS1524 (Bethyl, A300-001A), Rabbit anti-RAD51 (Santa, sc-8349). After washed by PBS, cover glasses were incubated for 40 min in 37 °C with secondary antibodies: Rabbit IgG F(ab′)_2_ fragment-Cy3 (Sigma) and goat anti-Mouse IgG-FITC antibody (Santa). After mounting with DAPI (VECTOR, H-1200), pictures were captured using an Olympus fluorescence microscope (BX 51) and analyzed with Image-Pro Plus. 100–200 cells were counted for quantitative immunofluorescence assay from three independent experiments.

For histological staining, freshly collected cervical tissues were embedded with optimal cutting temperature compound (O.C.T, Sakuraus, 4583) after using PBS clean, immediately frozen with liquid nitrogen and stored in -80 °C. Frozen tissue blocks were sectioned at 5 microns using microtome (LEICA, CM3050).

### Western blotting

Cells were resuspended in SDS sample loading buffer (pH8.0) and boiled for 5–8 min after radiotherapy and chemotherapy treatment. Nuclear extracts were separated through 8–15% SDS–PAGE, and then transferred onto PVDF membrane (Roche) which pre-soaked in methanol. Primary antibodies were diluted to the optimum concentration. HRP-conjugated anti-mouse or anti-rabbit IgG were got from DAKO. Blots were analyzed using Chemi docXRS (Bio-Rad). Antibodies used for immunoblotting included: anti-NBS1 phosphor-Serine 343 (Abcam, ab109453), anti-RPA32 phosphor-Serine 33 (Novus, NB100-544), anti-CHK2 phosphor-Threonine 68 (CST, 2348), anti-CHK1 phosphor-Serine 345 (CST, 2348S), anti-p53 phosphor-Serine 15 (CST, 9284S), anti-ubiquityl-histone H2B (Millipore, 05–1312), Tubulin (Sigma, T6074), anti-HA (Roche,11,867,423,001).

### Survival

Survival after homologous recombination repair defective target drug PARP inhibitor (Olaparib) treatments was assessed by clonogenic assays. Cells were collected and counted in a cell counting board. After plating of 600 cells in 6 cm dish, the plates were treated with Olaparib (0.5, 0.75, 1, and 1.5 μM). The cells were treated for 14 days for colony formation. Colonies were fixed by ice methanol and stained with Giemsa solution.

### Statistical analysis

SPSS 20.0 software was used to statistical analysis. All data came from three parallel assays. Student *t* test and one-way ANOVA was used to statistical significance analysis and the statistically significant difference was set at *P* < 0.05. Microsoft Office Excel 2007 was used for plotting.

## Results

### Increased DNA damage in HPV-positive pre-cancerous lesion

**Fig. 1:**
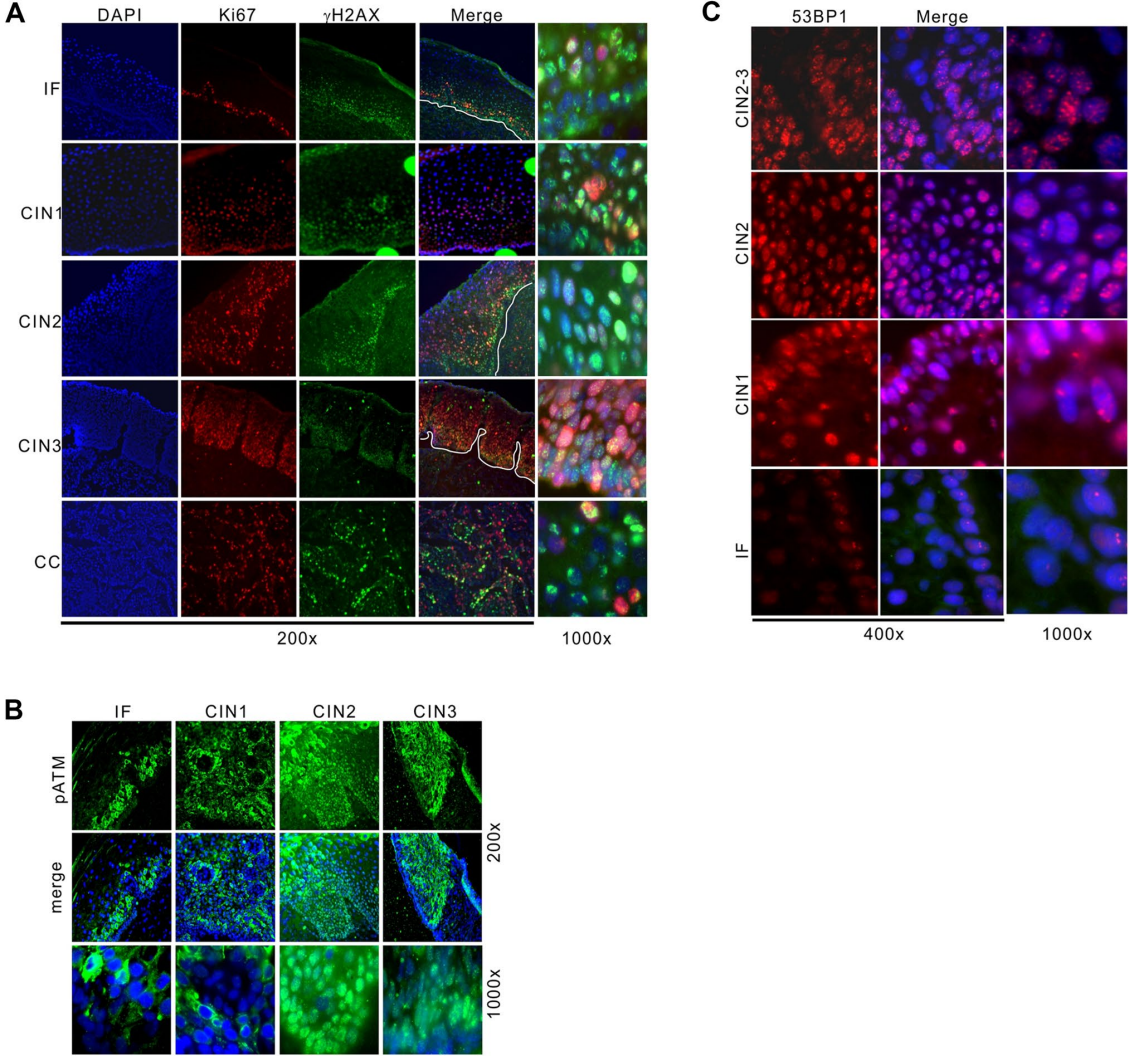
Elevated DNA damage in cervical epithelium. **A** Indirect immunofluorescent staining for Ki67, γH2AX in cervical epithelium. Nuclei were counterstained by DAPI. Stages of CIN and cervical cancer and magnifications (200 × and 1000x) are indicated. IF: inflammatory lesion. **B**, **C** As in (**A**), immunostaining for phosphorylated ATM (pS1981, **B**) and 53BP1 (**C**) in nuclear compartment of cervical epithelial cells. pS1981 displayed cytoplasmic staining in inflammatory lesions and CIN I, which we regarded as non-specific. All cervical samples were diagnosed as HPV16-positive according to pathological examination (see methods)

To investigate if DNA damage responses is aggravated in precancerous lesions of HPV-positive cervical epithelium, cervical tissues were biopsied and cryosectioned, and subjected to immunofluorescent staining. Subject tissues ranged from cervical inflammation, CIN (cervical intraepithelial neoplasia) stages I–III to cancer, all infected with high-risk subtype of HPV16 according to pathological diagnosis (Fig. 1A). Ki67 staining revealed active proliferation in cervical epithelium with inflammation, which expanded in CIN lesions to higher compartments of presumably differentiated cells. Cancerous samples displayed high Ki67 manifestation throughout tissues. Regarding the phosphorylation of H2AX, HPV + epithelium exhibited pan-nuclear or focal signals in the apical side, largely colocalized with Ki67 + compartment, indicative of abnormal activation of checkpoint. γH2AX signals exacerbated in CIN I–III, however, were restricted in cancer samples. This is reminiscent to previous indication that hyperactive checkpoint prevents precancerous cells from cancer initiation (Wang et al. 2015). Moreover, autophosphorylation of ATM kinase (ataxia telangiectasia mutated) that monitors various forms of DNA breaks was manifested in nuclei of CIN epithelial cells (Fig. 1B), suggesting unrepaired DNA lesions in these cells.

We also examined 53BP1 that mainly represents DSB repair. In inflammatory epithelium 53BP1 foci was low (1–2 per cell), while with the advancement of CIN stages it increased dramatically, especially in CIN II–III tissues (Fig. 1C). These evidences collectively demonstrate that in HPV + precancerous lesions, levels of DNA damage are elevated along with the disease progression of cervical intraepithelial neoplasia.

### HPV-E7 protein perturbs DNA damage responses

**Fig. 2:**
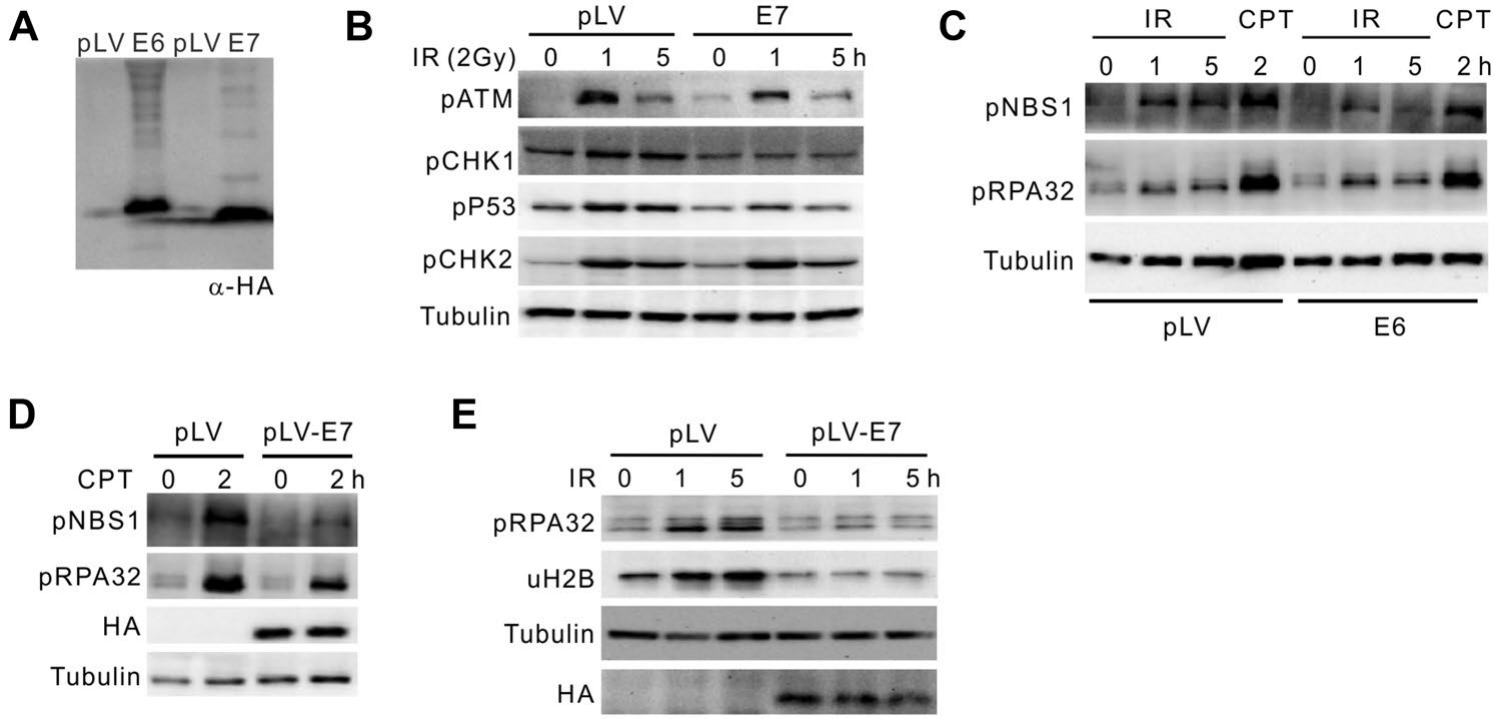
The HPV-E7 oncogene interferes with DDR. **A** Expression of HA-tagged E6 and E7 in HEK293T cells. pLV, empty vector. **B** Immunoblotting for ATM and ATR-dependent checkpoint phosphor-signals in 293T expressing E7 or not. Cells were challenged with IR (10 Gy) for indicated time. **C**–**E** Immunoblotting showing indicated phosphorylation of NBS1 and RPA32, or H2B monoubiquitination upon expression of E6 (**C**) and E7 (**C**, **D**). 293 T cells were insulted with CPT or IR (4 Gy) for indicated time. E7 expression was determined by α-HA immunoblotting

To evaluate the viral impact of HPV on DNA damage response, we synthesized and cloned HA-tagged E6 and E7 genes of HPV16 subtype, which were transfected into HEK293T cells in parallel with empty vector (Fig. 2A). In ionizing radiation (IR, 10 Gy)-treated cells, E7 expression did not greatly affect the ATM and ATR (ataxia telangiectasia-related)-dependent checkpoint activation regarding CHK1-pS345, CHK2-pT68, and p53-pS15 (Fig. 2C), except ATM-pS1981 that does not play crucial role in checkpoint activation. When cells carrying pLV or pLV-E6 plasmids were challenged with IR or Camptothecin (CPT, 2 μM) for 2 h, DNA repair proteins were phosphorylated as revealed by immunoblotting with α-pS343 for NBS1 and α-pS33 for RPA32 (Fig. 2B). However, these events representing activated homologous recombination were obviously decreased in the presence of E7. Further, histone H2B monoubiquitination (uH2B) is required for HR processing (Zeng et al. 2016). In E7-expressing cells, decreased RPA32 phosphorylation is accompanied by down-regulated uH2B level (Fig. 2B). We conclude that HPV-encoded E7 rather than E6 perturbs the activation of DNA damage repair, which potentially leads to accumulation of DNA lesions in HPV-infected cervical epithelium.

### E7 suppresses HR by perturbing the 53BP1-BRCA1 antagonism

**Fig. 3:**
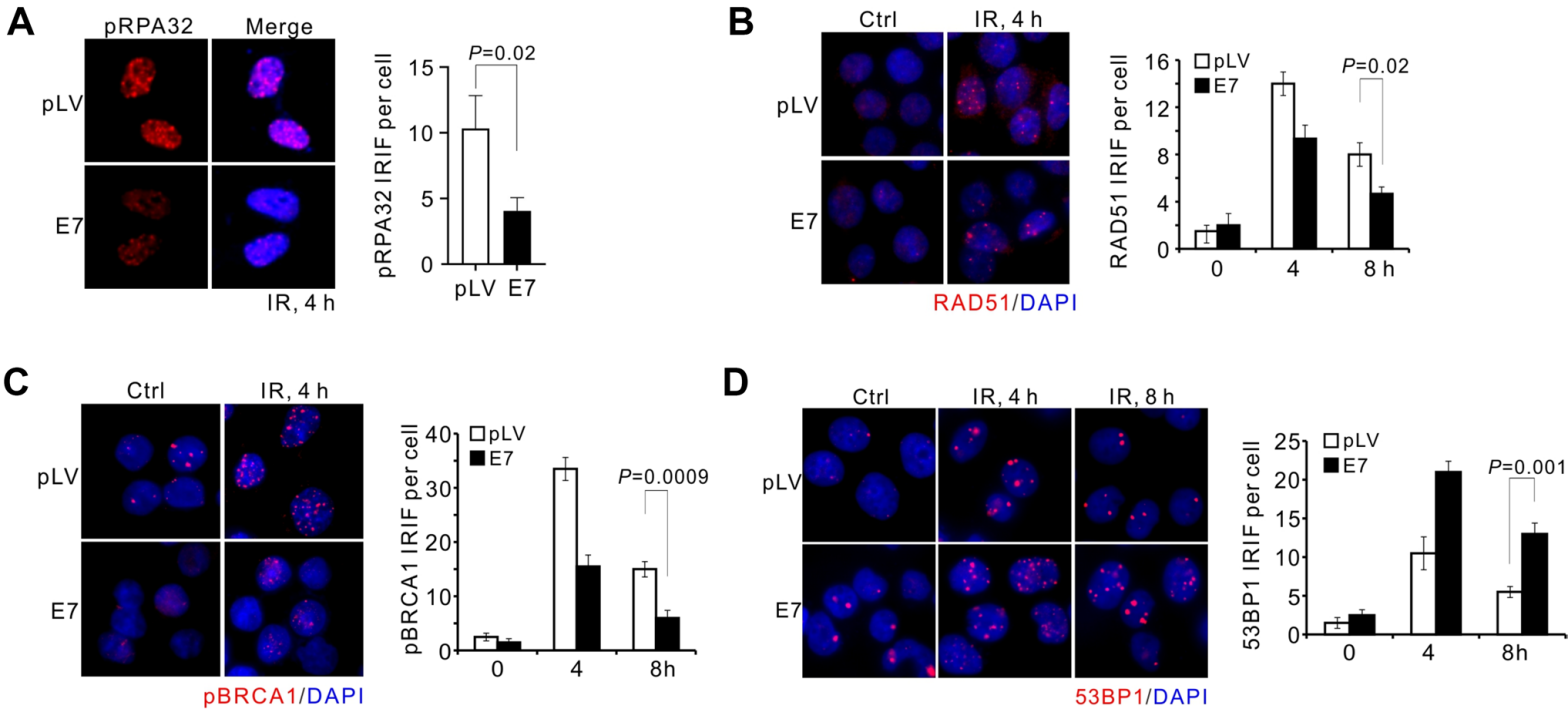
E7 Inhibits DNA end resection and HR in cervical cells. **A**–**D** Representative images (left) and quantification (right) for IR (4 Gy)-induced foci of RPA32-pS33 (**A**), RAD51 (**B**), BRCA1-pS1524 (**C**) and 53BP1 (**D**). E7 expression and IR recovery time are indicated. Cells in this figure were fixed with 4% PFA. *n* = experimental three replicates. Error bars indicate SD, *P* values by *t* test are shown for indicated groups

To strengthen the impact of E7 on DNA repair, we used indirect immunofluorescent staining to reveal the competency of DSB repair HPV-negative cervical cancer cells (C33A). Four hours after IR treatment RPA32-pS33 formed foci in C33A cells transfected with pLV vector, whereas the signal significantly decreased upon E7 expression (Fig. 3A). This indicated that E7 attenuated the production of single-stranded DNA at DSBs. Given ssDNA is required for commitment of homologous recombination, RAD51 foci is reduced in irradiated cells expressing E7 in comparison with control cells, corroborating the failure of HR in the presence of E7 (Fig. 3B).

HR is regulated by the pro-NHEJ factors (i.e. 53BP1) and pro-HR factors (i.e. BRCA1). The balanced occupancy of these factors determines the ssDNA generation, and following HR commitment. We examined the DSB recruitment of 53BP1 and phosphorylated BRCA1 in irradiated cells. When C33A cells expressing E7 are subjected to IR treatment, BRCA1-pS1981 formed less foci 4- and 8-h post-IR relative to cells transfected with empty vector (Fig. 3C). Inversely, more 53BP1 foci persisted till 8-h after IR treatment when E7 was expressed, indicating that E7 suppresses the function of BRCA1 but enhances the blocking effect of 53BP1 in ssDNA production (Fig. 3D). These results imply that HPV-E7 perturbs the balanced loading of counteractive 53BP1-BRCA1 at DSBs, thus disrupting the pathway choice of HR and NHEJ. This leads to a HPV-induced deficiency of homologous recombination repair.

### E7 renders cells susceptible to PARP inhibition

**Fig. 4:**
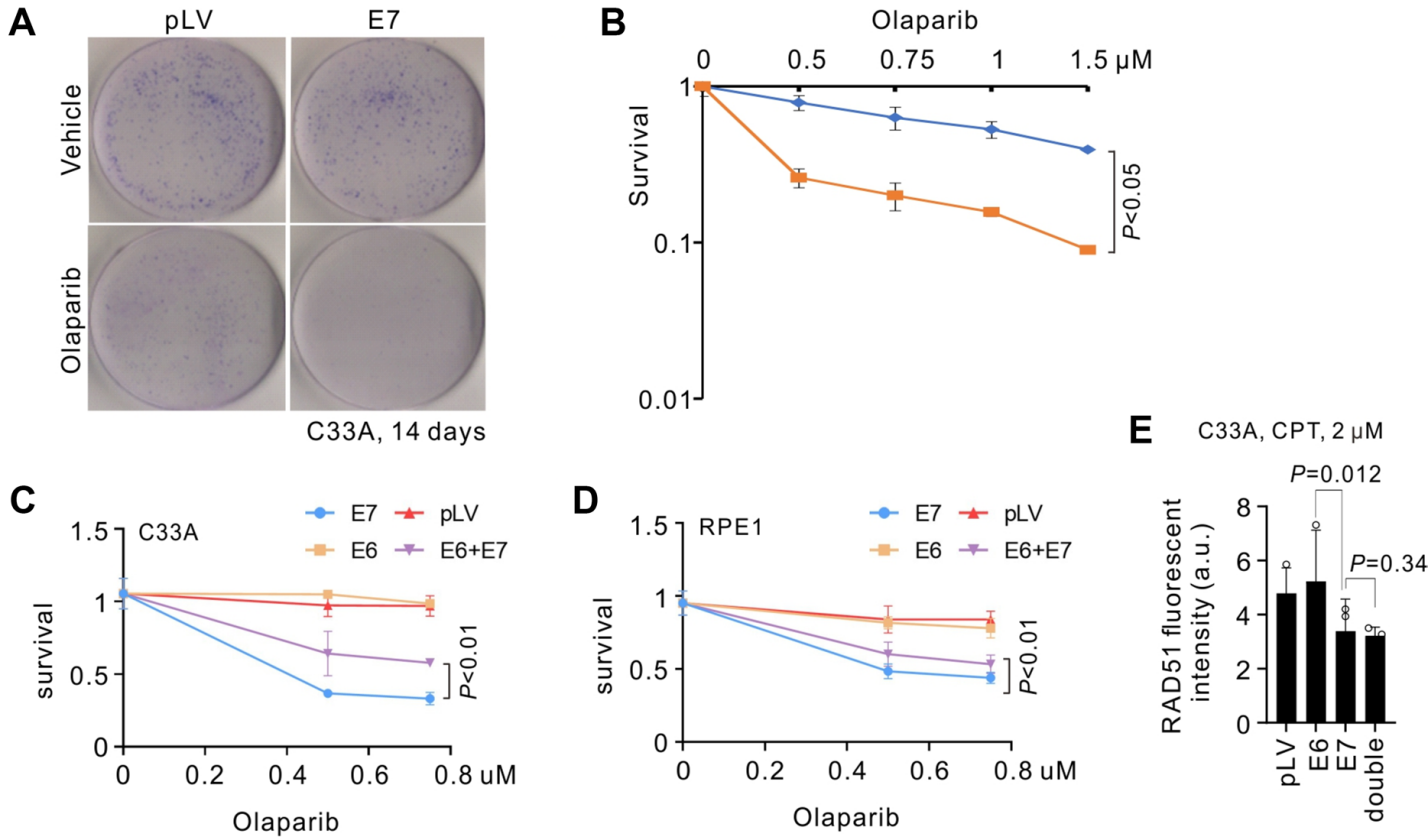
Synthetic lethality between E7-induced HRD and PARP inhibition. **A** Giemsa staining for colony formation of C33A cells with E7 expression or not. Olaparib was exposed for 14 days before PFA fixation. **B** Quantification for clonogenic experiment as in (**A**). Concentrations of Olaparib are shown. **C**, **D** Evaluation for PARPi sensitivity of C33A or RPE1-hTERT cells upon transfection with E6, E7 or both. Viability was measured with CCK8 at 96- or 72-h post-transfection for C33A or RPE1, respectively. For survival assays, *n* = 3 experimental repeats; error bars indicate SD; *P* values by *t* test are shown for indicated groups. **E** Quantification for immunofluorescent intensity of RAD51 in C33A cells expressing E6, E7 or both. Cells were permeabilized and fixed with methanol (see method), the treatment that depletes soluble proteins but leaves insoluble proteins chromatin-bound

The viral HRD in the presence of E7 suggests that HPV-bearing cells could be sensitive to PARP inhibitors due to lethal chromosomal aberrance caused by illegitimate end joining (Patel et al. 2011). To test the synthetic lethality of E7-indued HRD and PARPi, we expressed E7 in C33A cells in the presence of Olaparib or not, and evaluated cell viability by colony formation (Fig. 4A). Clearly, cells transfected with pLV-E7 plasmid failed to sustain the exposure of Olaparib, forming miniscule or dying colonies. In contrast, C33A transfected with E7 without Olaparib treatment survived well and proliferated robustly comparable to pLV empty vector. Quantitatively, C33A cells with E7 expression exhibited 60% less survival relative to those harboring pLV (Fig. 4B).

It is a paradigm that *TP53* gene is frequently mutated in cells carrying HRD including *BRCA1* mutation (Nik-Zainal et al. 2016). The loss of pro-apoptosis function of p53 can alleviate the cells death caused by DNA breaks in the absence of functional HR, thus circumventing the p53-dependent anti-cancer network. Similarly, p53 degradation induced by E6 may protect E7-induced HRD cells from cell death. To test if E6 can also antagonize PARPi sensitivity caused by E7, we expressed these proteins individually or concomitantly in C33A or RPE1-hTERT cells, the latter of which is a non-cancerous eye epithelial line. Apparently, co-expressing E6 with E7 alleviated PARPi sensitivity, however, failed to reverse it completely (Fig. 4C, D). The mitigating effect of E6 is attributed to the abrogation of pro-apoptotic activity of p53. E6 expression did not impact E7-induced HRD because no statistic difference was observed for chromatin-bound RAD51 between E7 alone and E7/E6 co-expression cells subjected to CPT challenge (Fig. 4E). Taken together, we conclude that HPV-E7 reduces the HR competence and resultant PARPi sensitivity. These defects are largely resistant to E6-dependent p53 destruction.

## Discussion

Homologous recombination is crucial in maintaining genome integrity. In many cancer types including BRCA1/2-mutated breast and ovarian cancer HR is impeded, causing high mutation burden and specific genomic imprints (Nik-Zainal et al. 2016). Besides BRCAness mutations in patients’ genome, DNA virus like hepatitis virus B can also causes HRD by disrupting ssDNA production required for HR repair, this drives the development of HBV-positive hepatocellular carcinoma (Ren et al. 2019). Here we report another virus-induced HRD type of carcinogenic process, where high-risk HPV-encoded factor E7 impairs DNA end processing and subsequent commitment of HR. As a common theme in cancer-related HRD, ssDNA processing and antagonism of BRCA1-53BP1 engagement at DSBs are often perturbed.

The HPV proteins E6 and E7 are crucial for viral activities and oncogenesis. It is interesting to note that E7-induced HRD is toxic to host cells and, therefore, disadvantageous for cancer development. However, this toxicity could be overcome by E6-dependent p53 degradation, which obviates checkpoint surveillance mechanism that kills the HRD cells. This is reminiscent of the co-existence of *TP53*/*BRCA1* mutations in breast cancer suggesting that p53 inactivation is a requirement for tumor progression in the setting of HRD (Na et al. 2019). However, while E6 protects infected epithelial cells from p53-driven apoptosis, it does not fully abrogate PARPi sensitivity induced by E7, nor rescue the RAD51 recruitment to damage sites (see Fig. 4C–E). Thus, we conclude that E6 helps circumvent the cellular toxicity of E7 and p53 during HPV infection and cervical cancer development, but cannot resist the PARPi sensitivity of infected cells.

It is possible that E7 has multiple impacts on DNA metabolism including encompassing DNA repair and oxidation. These effects may not be directly linked, but likely be attributed to the fundamental activity of E7 in DNA methylation. In this work, aberrant activation of γH2AX and 53BP1 prevailed in HPV16-positive CIN and cancerous samples, it may reflect either disturbed methylome that induce endogenous DNA breaks, or perturbation of methylation process during DSB processing in infected cells. Nevertheless, it is worthwhile to investigate the underlying mechanism of E7-induced HRD and methylation using primary keratinocytes or cervical organoid.

Our work reveals another cancer type potentially targeted by PARPi in addition to canonical BRCAness cancers carrying *BRCA1/2* mutations. We conclude that HPV-E7 is a potential driver for genome instability, providing an angle to understand its role in cancer development and a new route of targeted therapy.

## Data Availability

Participant data are available upon request from L.S. (drsongliang@163.com) under standard rules of data protection and ethical permissions.

## Abbreviations

HPV: Human papillomavirus
PARPi: Poly(ADP-ribose) polymerase inhibitor
ATM: Ataxia telangiectasia mutated
BER: Base excision repair
ATR: Ataxia telangiectasia-related
CIN: Cervical intraepithelial neoplasia
IR: Ionizing radiation
HRD: Homologous recombination defect
ssDNA: Single-strand DNA
CPT: Camptothecin
uH2B: H2B monoubiquitination
IF: Inflammatory lesion
RPA: Replication protein A
